# Transcriptional programs diverge in aging mouse and human skeletal muscle

**DOI:** 10.18632/aging.206382

**Published:** 2026-05-18

**Authors:** Charles D. Hwang, Siti Rahmayanti, Yori Endo, Seamus P. Caragher, Luisa Weber, Jessica Mroueh, Simone Marini, Indranil Sinha

**Affiliations:** 1 Division of Plastic and Reconstructive Surgery, Department of Surgery, Brigham and Women’s Hospital, Harvard University, Boston, MA 02115, USA; 2 Ludwig Maximilian University, Munich, BY 80539, Germany; 3 Department of Epidemiology, University of Florida College of Public Health and Health Professions, Gainesville, FL 32611, USA

**Keywords:** hypoxia, angiogenesis, aging, skeletal muscle, regeneration

## Abstract

Animal models provide a crucial scientific substrate for medical innovation, yet findings in these models do not always translate directly to humans. Although murine models are extensively employed to study skeletal muscle aging, the extent to which they diverge from the human aging process remains poorly understood. This study examined transcriptional changes with aging in mouse and human skeletal muscle. RNA bulk-sequencing was performed on gastrocnemius muscles from young and old C57BL/6 mice and compared to transcriptomic data from young and old healthy human vastus lateralis muscles obtained from the GESTALT study (NIA/NIH) via the Gene Expression Omnibus database. Cross-species comparison revealed substantial divergence in age-associated transcriptional profiles, with fewer than 5% of significant GO and KEGG terms shared between species. Hypoxia signaling, VEGFA, and inflammatory pathways showed concordant downregulation with aging in both species; however, angiogenesis, neurogenesis, and myogenesis demonstrated opposing or non-significant trends. These findings caution against direct extrapolation of murine aging transcriptomics to human skeletal muscle biology, though select conserved pathways may represent viable cross-species targets for future investigation.

## INTRODUCTION

Aging is associated with a progressive loss of skeletal muscle mass and function across multiple organisms [1, 2]. This can manifest as an inability to perform activities of daily living or maintain mobility [3]. Animal models have been developed to understand mechanisms underlying this aging-associated loss of muscle mass and identify potential therapeutic targets. Currently available animal models of aging, all of which exhibit loss of muscle and function to varying extent, include zebrafish [4], mouse [5], and rat [6]. Murine models, in particular, have been crucial in identifying potential therapeutic targets for improving skeletal muscle maintenance with aging [7]. However, although animal models provide a useful substrate for medical and scientific research, discordant findings have been commonly described in the existing literature between animals and humans at the transcriptional level [8]. A divergence in the transcriptomics between mouse and human have been described in embryogenesis [9], nephrogenesis [10, 11], neurological and cortical architecture and expression [12], macrophage stress responses [13, 14] and healthy myocardium [15, 16]. This may partially explain a poor rate of successful translation from bench to bedside. Given this, understanding whether the transcriptome diverges with aging in humans versus mice is critical in understanding the appropriateness of using a murine model of aging in skeletal muscle.

Previous evaluation of human muscle samples demonstrated a significant transcriptomic shift with aging in skeletal muscle, particularly in genes associated with cellular senescence and insulin signaling [17]. In contrast, similar studies in aging mouse models have highlighted increases in genes related to mitochondrial function and inflammation [18] and loss of hypoxia pathway signaling [5, 2] in muscle. Other tissue-types have demonstrated varying levels of similarities between the transcriptome with aging [19, 20]. With regards to skeletal muscle, specifically, Borsch et al. suggested that many pathways were conserved among mice, rats, and humans, up to the age of 70 years old with regards to the transcriptomic changes with aging [21]. The present study selected data from healthy human subjects aged 80 years and older, to provide a comparison point at an age where substantial muscle loss has occurred [22]. For comparison, 24- to 25-month-old mice were evaluated, as mice of that age similarly demonstrate a functional loss of mass and function and are similarly aged to 80-year-old humans with regards to lifespan [23].

## RESULTS

### Mouse and human muscles exhibit divergent transcriptomic phenotypes

NMR analyses of mice demonstrated significantly increased proportion of fat and commensurately decreased proportion of muscle and tetanic strength in the setting of similar total body weights (Supplementary Figure 1).

The transcriptional data were obtained from mouse gastrocnemius muscles through bulk-RNA sequencing performed on harvested muscles and from human vastus lateralis muscles through abstraction from NIH GEO. Raw counts were pre-processed with filters and normalization with resultant gene and count matrix files (Figure 1A). Volcano plots demonstrate genes with at least a 2-fold difference in expression levels (statistically significant) in mouse and human (Figure 1B). In gene ontology enrichment for biological processes, molecular functions, and cellular components, there were 1, 2, and 0 overlapping terms respectively. KEGG pathway database terms yielded 2 overlapping terms. These reflect less than 5% of the total number of statistically significant enriched keywords and pathways (Figure 1C).

**Figure 1:**
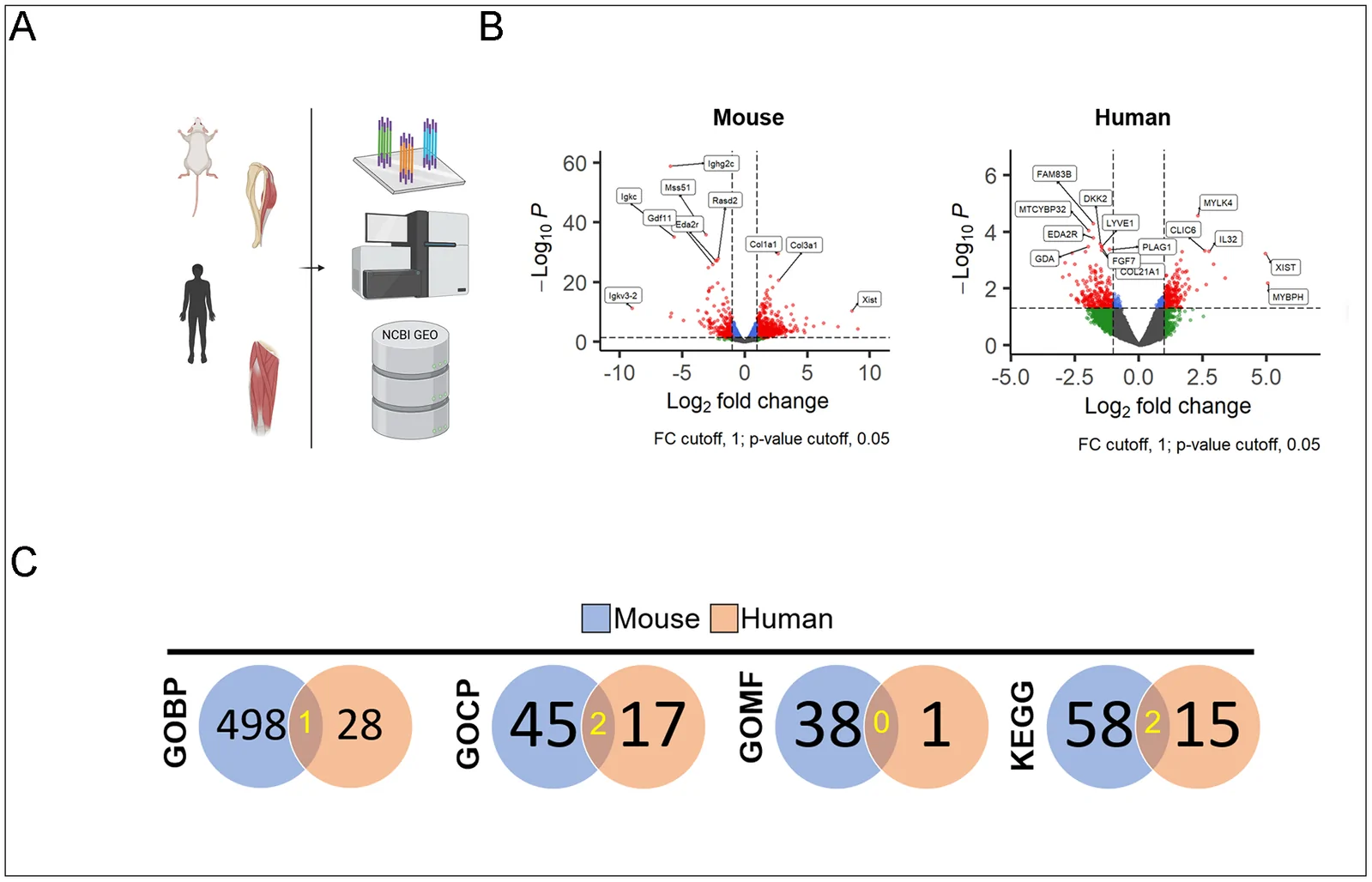
Schematic of mouse and human bulk-seq analysis and processing (**A**). Volcano plots demonstrate those with statistical significance and absolute value of fold change >1 (red) in mouse (left) and human (right) muscles (**B**). Counts of statistically significant enrichment terms derived from upregulated DEG in both old vs. young mice (blue circle) and humans (red circle) with the number of overlapping terms highlighted in yellow (**C**).

Mouse samples yielded an average of 25M reads per sample (n=6, min 23.3M, max 27.6M). 49315 differentially expressed genes in old vs. young comparison with 14472 matched ENSEMBL genes, 2760 genes with adjusted Benjamini–Hochberg FDR statistical significance (p.adj < 0.05). Human samples (n=13) yielded an average of 53M reads per sample (min 7.8M, max 118.8M). 57773 differentially expressed genes in old vs. young comparison with 21638 matched ENSEMBL genes, 613 genes with adjusted (Benjamini-Hochberg FDR) statistical significance (p.adj < 0.05).

### Mouse and human muscles exhibit heterogeneity in transcriptome with aging

Head-to-head comparisons of enrichment terms between species, stratified by the up- or down-regulation of transcription in young versus old. GO BP and KEGG terms are shown in Supplementary Table 1.

The 20 genes most upregulated with aging (expression increased in old muscle compared to young muscle) in mice and humans were visualized (Figure 2). Examination demonstrated little overlap between mouse and human. The two with similarities included osteocrin (*Ostn*; also known as musclin [24], a connective tissue derived humoral factor [25] with protective effects in setting of acute metabolic stressors on kidney [26] and muscle [27, 28] as well as ankyrin repeat domain-containing protein 1 (*Ankrd1*; also known as cardiac ankyrin repeat protein CARP) *Ankrd11p1*, a putative pseudogene resembling an ankyrin isotype involved in stress responses within skeletal muscle [29], as well as bony/cardiac development [30–33].

**Figure 2:**
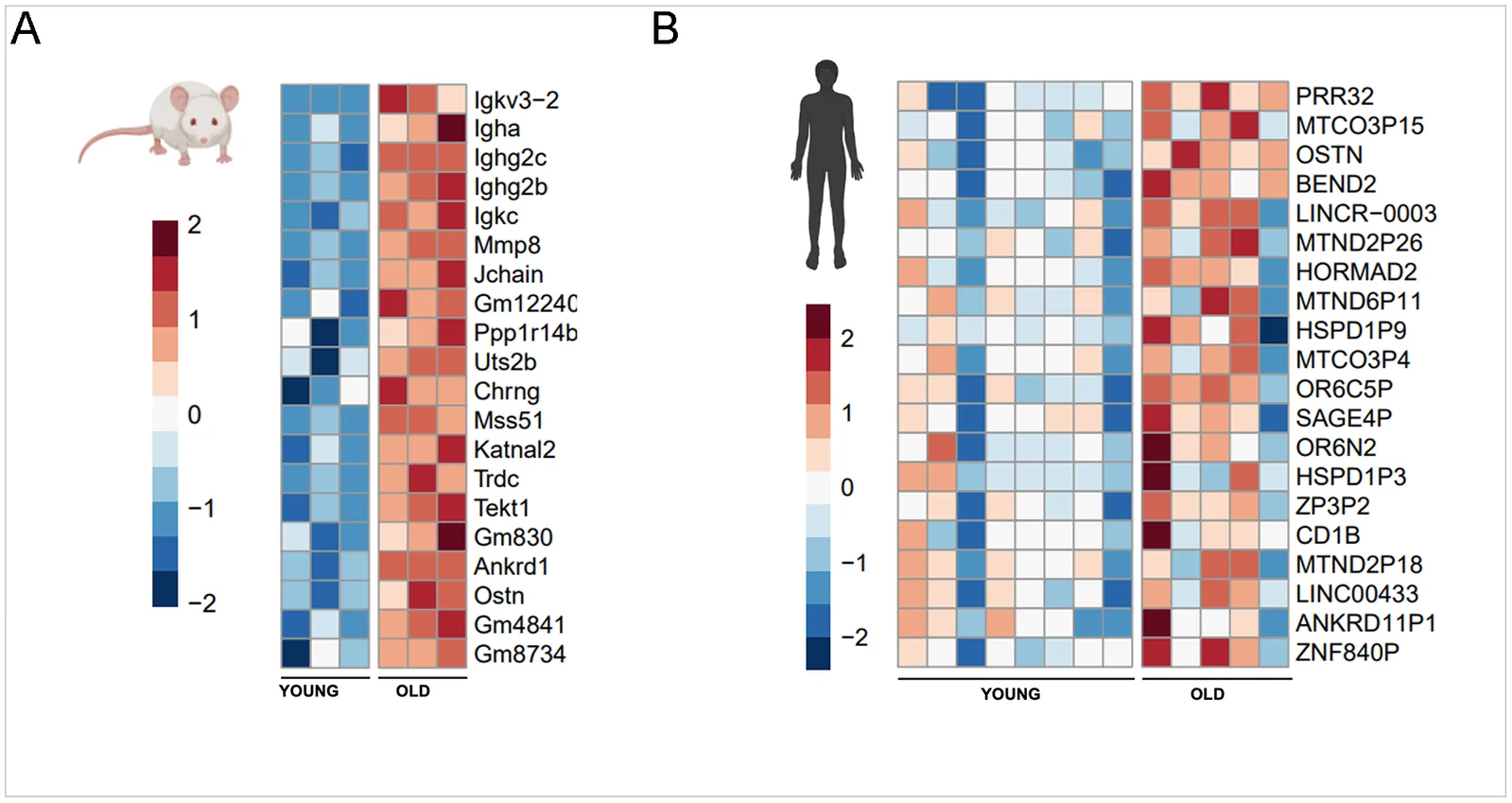
20 genes with greatest increase in expression with aging in mice (**A**) and human (**B**) muscle.

Between species, older mice demonstrated relative upregulation of inflammatory terms including several families of immunoglobulins and regulation of their binding/downstream responses (*Igkv3-2* [34]*, Igha* [35]*, Ighg2c* [36], *Ighg2b* [37]*, Igkc* [38], *Jchain* [39], *Ppp1r14b* [40]*, Trdc* [41]*, and Gm4841* [42]*.* In the human counterpart, a less unifying motif was found with various terms pertaining to modes of replication and transcriptional regulation via chromatin structuring (*BEND2* [43]*, HORMAD2* [44], long noncoding RNA modulation of Yap1 signaling (*LINCR-0003* [45], and zinc finger control of RNA polymerase II (*Znf840p* [46] along with various pseudogenes pertaining to stress response via heat shock proteins (*HSPD1P9, HSPD1P3)*.

Examination of the genes most differentially expressed in aging with lower expression in old muscle similarly limited overlap across species (Figure 3). Of note, X inactive specific transcript (*Xist* [47]) is found in the top 20 genes in both mouse and human, a long non-coding RNA that has been shown to have regulatory effects on the Wnt/β-catenin pathway in the proliferation of vascular smooth muscle cells, fibroblasts [47], and upregulation of the larger Hif-1a hypoxia axis in oncologic contexts [48–50].

**Figure 3:**
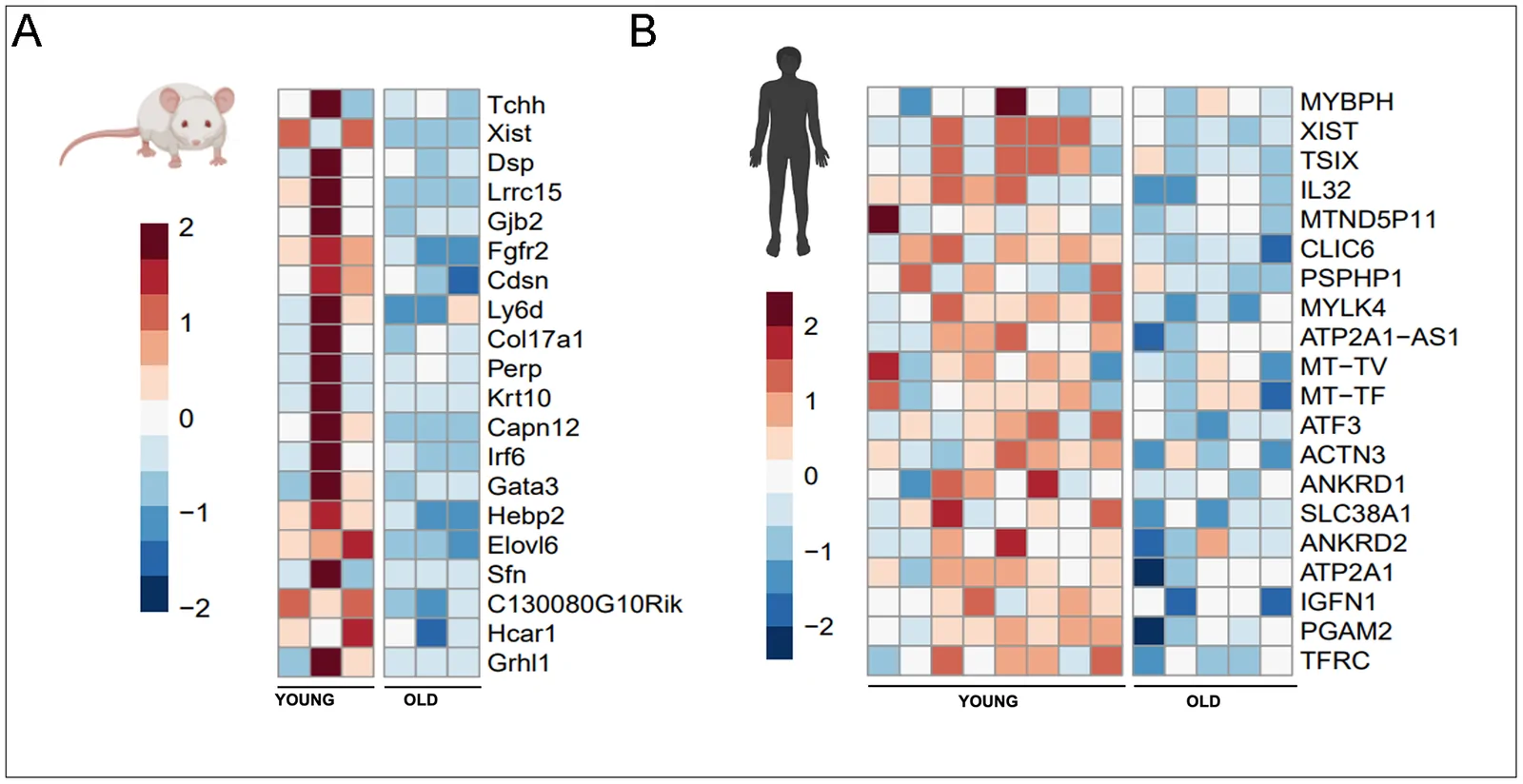
20 genes with greatest decrease in expression with aging in mice (**A**) and human (**B**) muscle.

In the mouse, other genes which exhibit less expression in old muscle include structural genes which govern cytoskeletal homeostasis (*Tchh* [50]*, Dsp* [51], *Hebp2* [52] and cell surface contact and receptor transduction for cellular proliferation/differentiation (*Lrrc15* [53], *Gjb2* [54], *Fgfr2* [55], *Col17a1* [56], *Perp* [57], *Krt10* [58], *Irf6* [59], *Gata3* [60], *Sfn* [61], *Hcar1* [62]. Of note, in humans *TSIX* [63] was found to be differentially expressed in young human muscle – a non-coding antisense transcript to *XIST* that results in its silencing in mouse tissues but conversely is found to potentiate human stem cell pluripotency [63]. The other genes specific to human include constituents of muscle (*MYBPH* [64], *MYLK4* [65]*, ACTN3* [66], *IFGN1* [67], various cytokines and regulators of inflammatory axis (*IL32* [68], *ATF3* [69–71]), and mitochondrial genes (*MT-TV [72], MT-TF [73]), TFRC* [74]. While *Ankrd1* was found upregulated in old mice, this same gene was found differentially upregulated in young humans. Another member of the ankyrin family, *Ankrd2* was also found similarly upregulated.

### Mice and humans demonstrate variable homology across hypoxia, vascular, neurogenic, and muscular programs

Given demonstrated transcriptome differences across age and species, we subsequently examined accepted gene lists for various pathways implemented into standard gene expression arrays (Qiagen, RT2 profiler PCR arrays) to further delineate the transcriptome patterns across species and age. In animal models, hypoxia signaling [2], VEGFA signaling [5, 75], as well as angiogenic capacity [76, 77] have been demonstrated to downregulate with aging muscle. Given previous works corroborating age related changes in nerve regeneration [78–80] and axonal transport [81], the neurogenesis pathway was also hypothesized to show an inverse relationship between the young and old. Outside of these extra-myogenic pathways regulating nascent muscle growth, aging has been also highlighted as a risk factor for impaired myogenesis at the myoblast and progenitor level [82, 83], implicating a reduced myogenic potential in aged muscle.

Principal component analysis (PCA) of the transcriptomic data highlighted notable differences in inter-sample variance between species. In mice, young (blue) and old (red) samples formed largely distinct clusters along PC1 (39% variance explained), indicating a clear and consistent age-related separation in gene expression (Supplementary Figure 2A). In contrast, the human PCA revealed considerable overlap between young and old samples, with PC1 accounting for 48.2% of variance yet failing to resolve the two age groups (Supplementary Figure 2B). This overlap reflects the inherently high biological variability present in human skeletal muscle transcriptomics, compounded by the disproportionate and modest sample sizes in this cohort (n=8 young, n=5 old). Factors such as sex, baseline fitness, and the broader genetic diversity of the human population likely contribute to this transcriptional heterogeneity, underscoring a fundamental challenge in cross-sectional human aging studies [83, 84]. Consequently, differential expression filtering and downstream heatmap visualization in mice were performed using a stringent FDR threshold of <0.1, while pairwise comparisons were assessed by Student's t-test. In humans, however, given that few genes achieved statistical significance after multiple testing correction, unadjusted p-values <0.05 were used to retain biologically relevant signal for visualization and pathway-level interpretation.

Visualization and pathway scoring revealed relative downregulation of the hypoxia and VEGFA pathways with age in both humans and mice (Figure 4A, 4B). Consistent with previous literature, angiogenesis and neurogenesis were also downregulated in aged mice; however, in humans, angiogenesis showed upregulation while neurogenesis did not reach statistical significance (Figure 4C, 4D). The myogenesis pathway showed no concordance between species (Figure 4E): while aged human patients demonstrated downregulation of the pro-myogenic transcriptome, this pattern was not recapitulated in mice at the ages tested. Finally, aging was associated with increased inflammatory and cytokine signaling in both species (Figure 4F).

**Figure 4:**
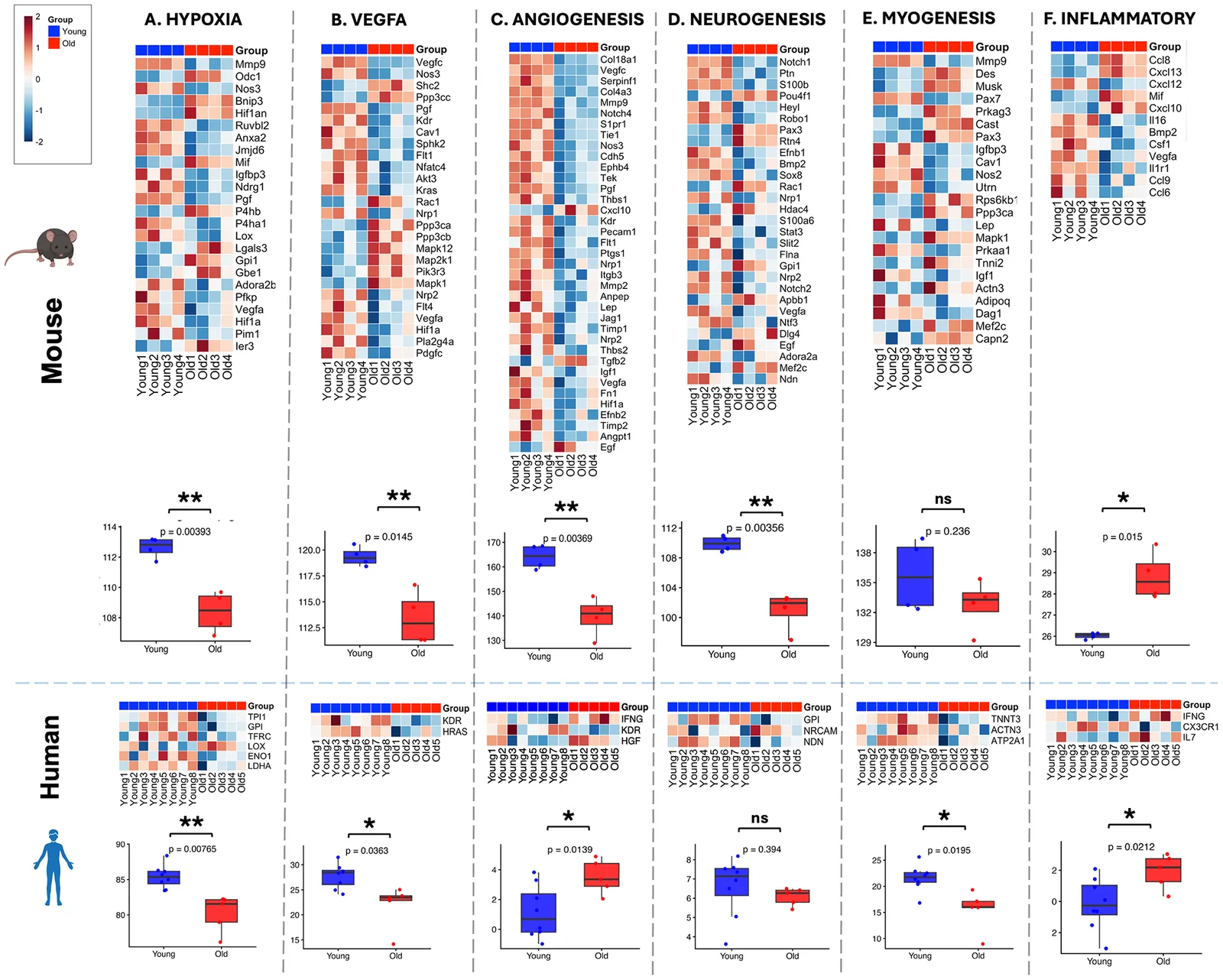
**Pathway gene expression during aging in mouse and human tissues.** Heatmaps display normalized logCPM expression of pathway genes. Differential expression in mouse samples was determined using edgeR with Benjamini–Hochberg correction (FDR < 0.1). In the human dataset, statistical significance was defined as p < 0.05 (unadjusted) due to high biological heterogeneity and limited sample size.

## DISCUSSION

Mice have long been accepted as an important model organism for studying the biological basis of human diseases given the high degrees of gene orthology between human and mouse (80% and 72%, respectively) [85], extensive annotations [86] and flexibility offered by genetic manipulations through Cre-lox transgenic systems and CRISPR Cas9 [87] technologies. However, similarities in genetic blueprint do not necessarily translate into transcriptional or translational concordance, which may impact the validity of biological inferences between mouse models and human disease [88]. In the present study, examination of RNA-Seq datasets from the gastrocnemius muscles of mice and vastus lateralis muscle of humans demonstrated divergent gene expression patterns in dimensional analyses across young and old age groups.

Consistent with prior report [88], human samples exhibited greater variance compared to mice. Volcano plot analysis revealed a larger number of genes with at least a 2-fold statistically significant difference in expression in mice relative to humans. Notably, muscles from young and old mice, which phenotypically exhibit decreased tetanic strength with age, displayed a distinctly binary transcriptional pattern. In contrast, human muscle samples demonstrated a more heterogeneous spectrum of gene expression profiles across age groups. Enrichment analysis of significantly upregulated genes revealed minimal overlap between species, with two or fewer shared terms across Gene Ontology categories and KEGG pathways. While mice on a uniform genetic background demonstrated clear age-dependent clustering, human samples were widely dispersed, suggesting substantial diversity in transcriptional states despite comparable age extremes.

To further investigate these differences, we performed direct cross-species comparisons of enrichment terms stratified by transcriptional directionality. In young mouse muscle, upregulated pathways reflected anabolic and structural processes, including extracellular matrix organization, cell migration, vascular and neural ingrowth, and stemness, relative to aged muscle. In contrast, young human muscle was characterized by enrichment of metabolic pathways, including pyruvate metabolism, glycolysis, oxidative phosphorylation, mitochondrial function, and muscle contraction (GO BP and KEGG terms shown in Supplementary Table 1).

Within human muscle, the most prominent age-associated decline involved metabolic pathways, which were significantly enriched in young compared to old samples. This aligns with established evidence that metabolism declines with aging [88], with skeletal muscle contributing to systemic metabolic dysfunction through impaired mitochondrial activity and insulin sensitivity [89]. These metabolic signatures were not significantly altered in mice, consistent with prior observations that fundamental differences in energy expenditure and metabolic regulation exist between species [90], potentially limiting translational relevance in this domain.

In contrast, murine muscle exhibited significant downregulation of pathways related to cellular proliferation and extracellular matrix (ECM) maintenance with aging [91]. Age-associated decline in muscle regenerative capacity has been extensively characterized in murine models [7, 92, 93], whereas direct evaluation in humans remains limited, though emerging *ex vivo* studies suggest similar trends [94]. Consistent with our findings, ECM composition and structural integrity decline with age in murine skeletal muscle, with preliminary evidence suggesting comparable changes in humans [95]. Given the role of the ECM as a reservoir for growth factors [96] and a regulator of muscle stem cell function [97], these alterations may directly influence regenerative capacity.

Cross-referencing enrichment analyses with the top differentially expressed genes further highlighted a consistent divergence in age-associated transcriptional programs between species. Notably, prior work comparing aging across mice, rats, and humans has reported greater transcriptomic similarity [21]. Several factors may account for these differences. First, muscle sampling differed between studies, with our analysis comparing gastrocnemius in mice to vastus lateralis in humans, whereas prior work utilized the same muscle group across species. Second, our human cohort included individuals over 80 years of age, in contrast to previous studies limited to individuals ≤70 years, potentially capturing a more advanced stage of transcriptomic remodeling associated with aging [17, 98].

Building on these observations, we examined several key regulatory pathways implicated in muscle aging, including VEGFA signaling, angiogenesis, neurogenesis, hypoxia response, inflammatory signaling, and myogenesis. Despite global transcriptomic divergence, select pathways demonstrated concordant age-related downregulation across species. Notably, while myogenesis did not reach statistical significance at the pathway level in mice, individual genes with established roles in muscle regeneration were differentially expressed, including Pax7 [99] and Igf-1 [100]. These findings suggest that, despite broad species-level differences, critical components of muscle aging biology remain conserved.

While the primary goal of this study was to characterize transcriptomic differences in healthy skeletal muscle aging between species, we acknowledge that therapeutic inference represents a valuable and clinically meaningful extension of these findings. The concordant downregulation of hypoxia and VEGFA signaling across both species supports the potential relevance of pro-angiogenic interventions in aged muscle, with agents targeting the HIF-1α axis and VEGF-mediated pathways warranting further investigation ([101, 102]). Similarly, the shared upregulation of inflammatory signaling across species lends biological rationale to anti-inflammatory strategies targeting the IL-6 and NF-κB pathways in the context of muscle aging [103, 104]. The divergence observed in neurogenic pathway expression between species, however, raises important questions regarding the direct translatability of pro-neurogenic approaches such as BDNF- or NT-3-based therapies from murine models to human aging muscle [105, 106]. These conserved pathways may represent viable cross-species therapeutic targets and should be explored in future pharmacological and interventional studies.

Several limitations should be considered. A fundamental distinction lies in genetic variability: human populations exhibit substantial inter-individual diversity, whereas inbred mouse strains are genetically homogeneous. This discrepancy likely contributes to the greater transcriptional heterogeneity observed in human samples and limits direct cross-species comparisons. The use of outbred mouse strains, such as CD-1 or CFW, may better model human population variability and should be explored in future studies [107]. Additionally, modest sample sizes, particularly in the human cohort (n=8 young, n=5 old), increase susceptibility to sampling bias despite rigorous preprocessing. Statistical thresholds also differed between species, with FDR < 0.1 applied in mice and unadjusted p-values used in humans due to limited significance after multiple testing correction.

Technical considerations further include the use of bulk RNA sequencing, which lacks cellular resolution and precludes identification of the specific cell populations driving observed transcriptional changes. Age-related shifts in cellular composition, including immune cell infiltration, may contribute to these differences. Additionally, variation in muscle type between species may influence transcriptomic profiles [108]. Finally, as a cross-sectional study, these analyses capture a single timepoint and do not reflect longitudinal transcriptional dynamics across aging.

Despite these limitations, the partial convergence of key aging-associated pathways supports the continued utility of murine models in studying skeletal muscle aging. However, our findings emphasize the need for careful interpretation of cross-species transcriptomic data and underscore the importance of integrating human datasets to validate and contextualize preclinical observations.

## MATERIALS AND METHODS

### Animals

All animal procedures were approved by the Institutional Animal Care and Use Committee (IACUC) of Brigham and Women's Hospital (Protocol number: 2016N000375) and were performed in accordance with the U.S. National Institutes of Health guidelines. 20-22-week-old young (n=4) and 24-25-months old (n=4) C57BL/6 mice were obtained from Jackson Laboratories and the National Institution of Aging, respectively. The animals were housed at the Brigham and Women’s Hospital Animal Care Facility following a 12 light / 12 dark cycle and were given ad libitum access to food and water.

### Murine nuclear magnetic resonance imaging

Body composition, whole body fat mass, lean body mass, free water and total body water were measured, prior to sacrifice, by nuclear magnetic resonance (NMR) using the EchoMRI-100™ QMR system (Houston, TX) as previously described [109].

### Murine *in situ* force testing

*In situ* force tests were performed prior to muscle sacrifice and following NMR testing as previously described [110]. Briefly, the right gastrocnemius (GA) muscle and Achilles tendon were isolated. The tendon was then transected at the distal most point of insertion and then sutured onto the lever arm of the force transducer (Aurora Scientific, Aurora, Ontario, Canada). Direct stimulation of the muscle was performed with needle electrode placement within the muscle. The tetanic force was measured with pulses of 100 Hz with increasing amperage from 10mA to 1A. The maximum tetanic force of the right GA muscle of each animal was normalized to the estimated cross-sectional area (CSA) of the muscle (mm^2^) calculated as CSA = Muscle weight (mg)/[1.06×Lo(mm)].

### RNA Bulk-sequencing analysis

Gastrocnemius muscle was harvested from the animals and immediately snap-frozen using liquid nitrogen for the subsequent analyses. Total RNA was extracted using TRIzole reagent. Following rRNA deletion, the sequencing library was prepared using Illumina HiSeq^®^ at 2x150bp configuration, with the minimal read of 30 million base pairs per sample. After quality assessment and adaptor trimming, the reads were aligned to the reference genome using STAR [111]. The expression level of each gene was then estimated by counting the number of reads that aligned to each exon using FeatureCounts [112] and normalized as fragments per kilobase per million reads (FPKM) and log_2_ transformation. Resulting matrix files were further filtered for lowly expressed genes and gene inclusion criteria 10/L ratio as described by Chen et al. [113]. Differential expression analysis was performed using edgeR [113] to compare the gene expression in the murine old and young groups (database available in GEO database: GSE155937 [114]. Sample similarity was assessed by dimensional reduction via biological coefficient of variance (BCV) distances.

### GEO database comparative analysis and pathway scoring

Data from healthy human vastus lateralis muscles (young, 22-30 years, n=8; old, 80-83 years, n=5) were derived from the GESTALT study (NIA/NIH) as found in the Gene Expression Omnibus (NIH) database (GSE164471, [17]). Differential expression profile was determined via edgeR as described above.

Known QIAGEN microarray genesets were investigated (Qiagen, RT2 profiler PCR arrays): Hypoxia (PAMM-032Z), Vegf (PAMM-091Z, AHS-091Z), Angiogenesis (PAMM-024Z, PAHS-024Z), Neurogenesis (PAMM-404Z, PAHS-404Z), Myogenesis (PAMM-099Z, PAHS-099Z), and Inflammatory Cytokines and Receptors (PAMM-011Z, PAHS-011Z). Normalized counts across all genes were summed for each sample or patient to generate a representative pathway score. Young and old cohorts were compared using Student’s t-test, with significance thresholds set at FDR < 0.10 (Benjamini-Hochberg correction) for mouse and unadjusted p < 0.05 for human samples, the latter reflecting the high inter-individual biological variability inherent to human cohorts.

### Gene identification and enrichment/ontology

Gene annotations performed with AnnotationHub (Bioconductor) via mouse (AH98078, Ensembl 105 database for Mus musculus) and human (AH98047, Ensembl 105 database for Homo sapiens) libraries. Agnostic gene enrichment and ontology analyses were performed with Enrichr [115], utilizing Gene Ontology (BP2021, CC2021, MF2021) and KEGG pathway libraries (mouse 2019, human 2021).

## Supplementary Material

Supplementary Figures

Supplementary Table 1
