# Supplementary material for: Transcriptional programs diverge in aging mouse and human skeletal muscle
Source: Aging (Albany NY). 2026 May 18;18(1):575–92. doi: 10.18632/aging.206382 (PMC13632859; doi:10.18632/aging.206382)
Supplement: Supplementary file 1 [file aging-18-1-206382-s001.pdf]

SUPPLEMENTARY FIGURES

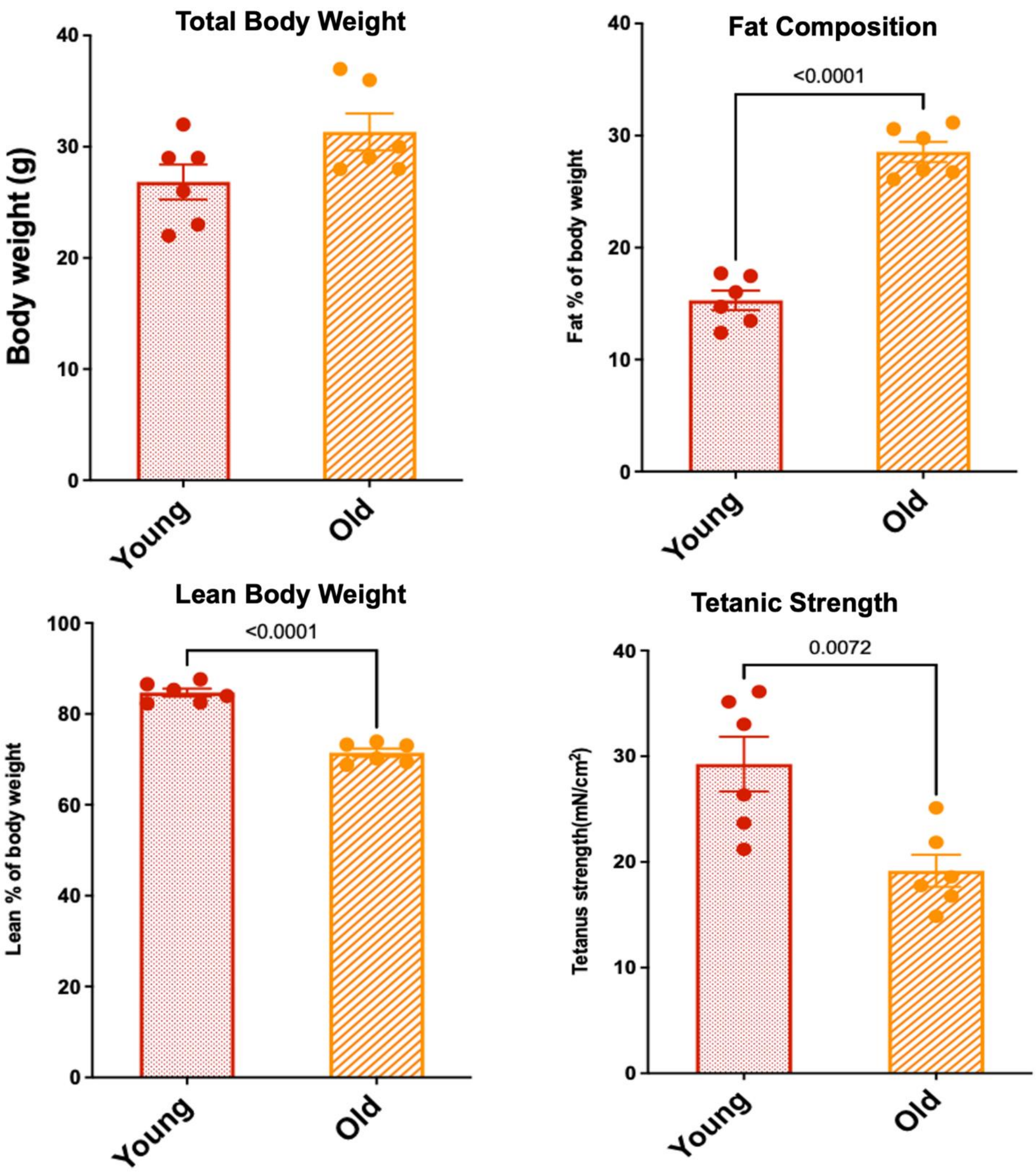

Supplementary Figure 1. Schematic of phenotypic analyses on murine Nuclear Magnetic Resonance (NMR) imaging (**A**–**C**) and *in situ* force testing (**D**). Young vs old mice in comparison of (**A**) total body weight (**B**) fat composition (**C**) lean body weight (**D**) tetanic strength.

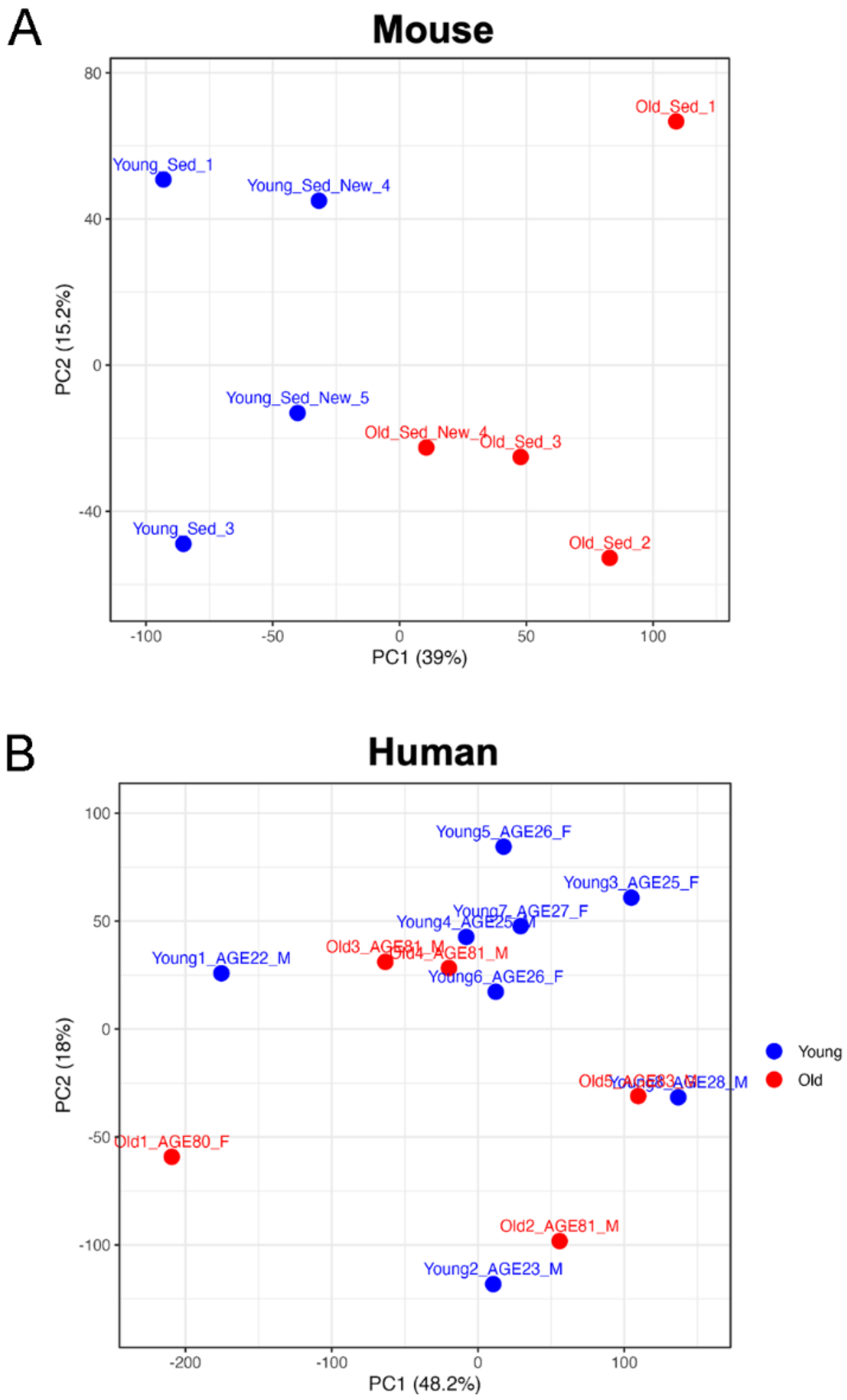

C

PCA Label	Heatmap Label	Species
Young_Sed_1	Young1	Mouse
Young_Sed_3	Young2	Mouse
Young_Sed_New_4	Young3	Mouse
Young_Sed_New_5	Young4	Mouse
Old_Sed_1	Old1	Mouse
Old_Sed_2	Old2	Mouse
Old_Sed_3	Old3	Mouse
Old_Sed_New_4	Old4	Mouse
Young1_AGE22_M	Young1	Human
Young2_AGE23_M	Young2	Human
Young3_AGE25_F	Young3	Human
Young4_AGE25_M	Young4	Human
Young5_AGE26_F	Young5	Human
Young6_AGE26_F	Young6	Human
Young7_AGE27_F	Young7	Human
Young8_AGE28_M	Young8	Human
Old1_AGE80_F	Old1	Human
Old2_AGE81_M	Old2	Human
Old3_AGE81_M	Old3	Human
Old4_AGE81_M	Old4	Human
Old5_AGE83_M	Old5	Human

Supplementary Figure 2. Principal component analysis and sample label correspondence. (**A**) PCA of mouse skeletal muscle RNA sequencing data (young, blue, n=4; old, red, n=4) showing clear age-group separation along PC1 (39% variance explained). (**B**) PCA of human skeletal muscle data (young, blue, n=8; old, red, n=5) demonstrating substantial inter-sample overlap along PC1 (48.2% variance explained), reflecting high inter-individual biological variability. (**C**) Sample label correspondence between PCA and heatmap visualizations in Figure 4.
